# The Regulation of LexA on UV-Induced SOS Response in *Myxococcus xanthus* Based on Transcriptome Analysis

**DOI:** 10.4014/jmb.2103.03047

**Published:** 2021-05-24

**Authors:** Duo-hong Sheng, Ye Wang, Shu-ge Wu, Rui-qin Duan, Yue-zhong Li

**Affiliations:** State Key Laboratory of Microbial Technology, Institute of Microbial Technology, Shandong University, Qingdao 266237, P.R. China

**Keywords:** *M. xanthus*, LexA, SOS response, UV-C irradiation

## Abstract

SOS response is a conserved response to DNA damage in prokaryotes and is negatively regulated by LexA protein, which recognizes specifically an “SOS-box” motif present in the promoter region of SOS genes. *Myxococcus xanthus* DK1622 possesses a *lexA* gene, and while the deletion of *lexA* had no significant effect on either bacterial morphology, UV-C resistance, or sporulation, it did delay growth. UV-C radiation resulted in 651 upregulated genes in *M. xanthus*, including the typical SOS genes *lexA*, *recA*, *uvrA*, *recN* and so on, mostly enriched in the pathways of DNA replication and repair, secondary metabolism, and signal transduction. The UV-irradiated *lexA* mutant also showed the induced expression of SOS genes and these SOS genes enriched into a similar pathway profile to that of wild-type strain. Without irradiation treatment, the absence of LexA enhanced the expression of 122 genes that were not enriched in any pathway. Further analysis of the promoter sequence revealed that in the 122 genes, only the promoters of *recA2*, *lexA* and an operon composed of three genes (*pafB*, *pafC* and *cyaA*) had SOS box sequence to which the LexA protein is bound directly. These results update our current understanding of SOS response in *M. xanthus* and show that UV induces more genes involved in secondary metabolism and signal transduction in addition to DNA replication and repair; and while the canonical LexA-dependent regulation on SOS response has shrunk, only 5 SOS genes are directly repressed by LexA.

## Introduction

Genomic DNA is the most important cellular component of bacteria, and its stability is related to bacterial life, death or mutation. However, DNA is vulnerable to a variety of environmental factors such as heat, acidity, dryness, radiation, alkylating agents, free radicals, etc. In response to these DNA threats, bacteria have evolved a conserved inducible DNA damage protection and repair mechanism termed SOS response [1, 2]. The canonical SOS response is known to be LexA/RecA dependent. LexA protein binds specifically to an imperfect palindrome sequence named SOS box (LexA box), which is located on the promoter of SOS genes and blocks their expression. Upon DNA damage, RecA polymerizes along the exposed single-stranded DNA, forming activated nucleoprotein filaments to activate the self-cleavage (autoproteolysis) of LexA, thereby releasing the expression of SOS genes [2-5]. LexA-dependent SOS response is a unique regulation mode of DNA damage stress response in bacteria and is highly conserved throughout the bacterial domain, making it a potential antibiotic screening target [4-7].

In addition to the ubiquitous LexA-dependent SOS response, LexA-independent pathways have also been found in bacteria to control SOS response [8, 9]. For example, in *E. coli*, the β-lactam antibiotics that inhibit cell-wall synthesis can induce SOS response via a pathway involving the DpiBA two-component signal transduction system [10, 11]. Similarly, *E. coli* stationary-phase sigma factor RpoS also positively controls the expression of SOS-regulated *dinB* gene against β-lactam antibiotics [10-12]. Moreover, *Acinetobacter baylyi* lacks the *lexA* gene and the SOS response can be induced in a LexA-independent pathway to activate the DNA repair, in which protein UmuDAb performs like a LexA-like repressor functioning on a sub-set of DNA damage-induced genes [12, 13].

Genome-scale approaches have greatly improved our overall understanding of the SOS genes induced by DNA damage in both LexA-dependent and -independent modes. The number and types of genes regulated by LexA have been found to vary extensively among bacteria. For instance, in UV-irradiated *E. coli* cells, 168 genes were upregulated of their expression levels, of which 43 genes were directly dependent upon the LexA regulation [14], including genes involved in DNA replication and repair. In the gram-positive model bacterium *Bacillus subtilis*, the LexA regulon comprises 33 genes among which only eight are homologous counterparts of the *E. coli* SOS genes [15]. Comparison of the SOS regulons among various bacterial species and genera allowed the description of LexA-regulated core regulons comprising *recA*, *uvrA*, *lexA* and *recN*, which might correspond to the ancestral core set [16]. In addition, some genes have been found to be regulated *lexA*-dependently and -independently [10, 17]. So far, there is no reliable explanation for the evolution and relationship between the manner of LexA-dependent and -independent SOS induction.

Myxobacteria are unique among bacteria, being characterized by gliding motility, predation, multicellular fruiting bodies, prolific production of secondary metabolites, and a large genome with many gene duplications. *Myxococcus xanthus* DK1622 is the model strain of myxobacteria. Previous studies have reported that SOS response in *M. xanthus* could be induced by DNA damage agents (naphthenic acid, mitomycin C or UV ray) [18-20], but has not been analyzed at the whole genome level. *M. xanthus* contains one *lexA* gene and two *recA* genes in its genome and RecA1 could facilitate LexA self-cleavage [20]. However, some conserved SOS genes in *M. xanthus*, including *recA*1, *uvrA* or *recN*, have no SOS box on their promoters [18]. In order to clarify the SOS response and LexA-dependent regulation in *M. xanthus*, we compared the transcriptional profiles between the wild type and *lexA* deletion mutant with UV-C irradiation or not. It was found that UV-C irradiation induced a large number of genes, which were mainly enriched in pathways of DNA replication and repair, metabolism and signaling, and only 5 UV-induced genes, including *recA*2, *lexA*, *pafB*, *pafC* and *cyaA*, were regulated by LexA directly.

## Materials and Methods

### Bacterial Strains and Growth Conditions

Strains, plasmids, and oligonucleotides used in this study are listed in Tables S6 and S8. The *M. xanthus* strains were cultivated in Casitone-based CYE medium (10 g/l casitone, 5 g/l yeast extract, 10 mM MOPS and 4 mM MgSO_4_, pH 7.6) for the growth assay or on TPM agar plate for the development assay. *E. coli* strains were routinely grown on Luria-Bertani (LB) agar or in LB liquid broth (10 g/l peptone, 5 g/l yeast extract, and 5 g/l NaCl, pH 7.2). *M. xanthus* strains were incubated at 30°C, and *E. coli* strains were at 37°C. When required, a final concentration of 33 μg/ml of kanamycin (Kan) or 100 μg/ml of ampicillin (Amp) was added to the solid or liquid media.

### Gene Deletion Mutation

**Fig. 1:**
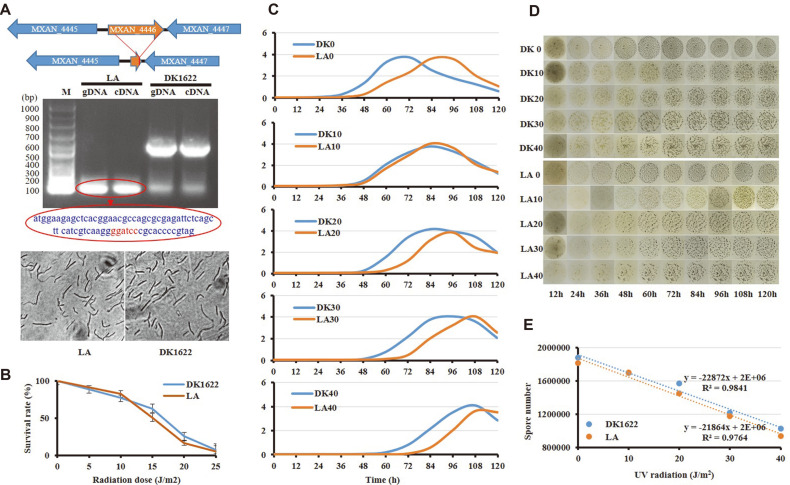
Deletion mutation analysis of the *lexA* gene. (**A**) Construction and verification of the *lexA* deletion mutant; (**B**) Survival analysis with different doses of UV-C irradiation in DK1622 and LA; (**C**) Growth analysis of DK1622 and LA with different doses of UV-C irradiation (0 ~ 40 J/m^2^); (**D**) Fruiting body formation with different doses of UV-C irradiation; (**E**) Statistical analysis of the sporulation abilities of DK1622 and LA.

Markerless mutation was carried out to knockout the *lexA* gene in *M. xanthus* DK1622 using pBJ113 plasmid. The plasmid contains a kanamycin-resistant cassette for the first round of screening and a *galK* gene for the negative screening [32, 33]. To avoid the potential effect on the downstream gene expression, we deleted the middle part of the *lexA* gene and left the N terminal 30-bp and C terminal 9-bp fragments (Fig. 1A). Briefly, both the upstream and down stream homologous arms were amplified with primers (listed in Table S7) and ligated at the BamH1 site. The arm sequences were inserted into the EcoRI/HindIII site of pBJ113. The resulting plasmid was introduced into *M. xanthus* strains via electroporation (1.25 kV, 200 W, 25 μF, 400Ω, 1 mm cuvette gap). The second round of screening was performed on CYE plates containing 1% galactose (Sigma). The *lexA* mutant (named LA) was identified and verified by PCR amplification and sequencing.

Restriction enzymes, DNA ligase, and other enzymes were used according to the manufacturers’ recommendations. All DNA products were validated by DNA sequencing.

### Growth, Mutation Rate and Sporulation Assays

*M. xanthus* cells in exponential phase were used for determining the cell growth curves. For UV irradiation, the cell suspension (15 ml) in 130 mm glass Petri dishes was stirred gently with a magnetic rod and irradiated with UV ray (UV-C, 254 nm) at room temperature. Then, the cells were diluted in fresh medium and incubated at 30°C. The density of the cells was measured every 12 h to generate the growth curves.

The mutation rate assay was conducted by screening the nalidixic acid-resistant strains according to the previous report [34]. Briefly, approximately 5 × 10^9^ cells of *M. xanthus* were placed on CYE agar containing 40 μg/ml nalidixic acid. Nalidixic acid-resistant candidates were counted and mutation rate was calculated using the following formula: mutation rate = nalidixic acid resistant clones / 5 × 10^9^.

Sporulation experiments were performed on TPM agar plates [35]. Ten microliters of (10^7^ cells/ul) cells were dot-inoculated on TPM agar plate and allowed to develop at 30°C. Microscopic examination of cell aggregation and fruiting body formation were conducted every 12 h. Spores were counted after five days as follows: five colonies were collected and serially diluted in liquid CYE medium and myxospores were resuspended by sonication at 200 W for 10 s twice. The suspensions were incubated at 55°C for 2 h to kill the vegetative cells and then used for CYE plate numbering.

### RNA Extraction, RT-PCR and RNA Sequencing Assay

Total RNA of *M. xanthus* cells was extracted using RNAiso Plus reagent (Takara, China) following the manufacturer’s protocol. RNA concentration was quantified by Nanodrop 2000 (Thermo Fisher Scientific, USA). Genomic DNA (gDNA) in the RNA sample was removed with gDNA Eraser (Takara). The first-strand cDNA was synthesized with random primers by using the PrimeScript RT Enzyme Reagent Kit (Takara) following the manufacturer’s instructions.

Synthesized cDNA samples were diluted 5 times prior to RT-PCR. The primers designed for *imuA*, *dnaE2*, *recA*1 and *recA*2 are listed in Table S7. RT-PCR was accomplished using the SYBR Premix Ex Taq Kit (Takara) on an ABI StepOnePlus Real-Time PCR System (Thermo Fisher Scientific), following the program: 3 min at 95°C, followed by 40 cycles of 30 s at 95°C, 30 s at 55°C, and 15 s at 72°C. The relative quantification of mRNAs of interest was performed by the comparative CT (2^−ΔΔCT^) method, with the *gapA* gene, encoding for glyceraldehyde-3-phosphate dehydrogenase, as an endogenous control, as previously described (Peng et al., 2017).

RNA sequencing was conducted by Illumina platform, and the purified double-stranded cDNA was end-repaired, added with A bases, and ligated with sequencing adaptors. After PCR amplification, the cDNA was sequenced on an Illumina HiSeq 4000 (Novogene, China). Three independent repeats were tested for each strain, and the quality information of sequencing for the total 12 samples is shown in Fig. S4. All the upregulated and downregulated genes were obtained by comparing with control, and their gene functions were explored using database annotations such as GO and KEGG. A *p*-value of < 0.05 was used as the threshold to identify differential expression genes (DEGs).

The level of mRNA expression was normalized by FPKM (fragments per kilobase of transcript per million mapped reads). Genes with a log_2_FoldChange ≥ 0.4 and *p* < 0.05 were considered as DEGs.

### Protein Expression, Purification and Gel Shift Analysis

*LexA* gene was cloned into *E. coli* expression vector pET15b. Then, the recombinant plasmids were transformed into *E. coli* BL21(DE3) and cultured in LB medium at 37 °C. Induction of expression was initiated at OD_600_ = 0.6 by the addition of IPTG (1 mM). Cells were harvested 3 h later and sonicated. From crude extracts, the 6xHis-tagged proteins were purified using Ni-NTA agarose columns (Qiagen) according to the manufacturer's instructions.

Gel shift analysis was used to evaluate the binding ability of LexA protein to the SOS box sequence. The SOS box sequence from the primers of genes *recA2* (TTTTTTTCGCGTACTAAAAGCGCGTTCAGGTGAGC), *lexA* (TTTTTTGTTGACTCTACACGTCTGTTCAGGGAGAG), *recN* (TTTTTTGTCGGACCTGCTAGGGTGTTC AGGTACCA), *pafBC* (TTTTTTTGATTACCTGCTTGTTTGTTCAGGCGCGT), were synthesized in (TSINGKA) and labeled with biotin at the 5’ end. Then, 25 μl reactions containing 25 mM Tris-HCl, pH 7.0, 50 mM NaCl, 4%glycerol, 1 mM DTT, 2 μg protein and 25 μmol DNA were incubated at 32°C for 20 min, and analyzed by gel electrophoresis in 0.8% agarose gels. Migration was performed for 2 h, at 2 V/cm and 4°C, and then transferred to a nylon membrane. Blotting was performed using a Bio-Rad electro-blotting system (model Trans blot) according to the manufacturer's instructions. In addition, the biotin-labeled DNA was detected by LightShift Chemiluminescent EMSA Kit (Thermo) according to the instruction manual. Three independent experiments were performed, and a representative sample is shown.

## Results and Discussion

### Absence of *lexA* Delayed the Growth, But Did Not Affect UV-C Resistance and Sporulation of *M. xanthus*

There is a single *lexA* gene in the genome of *M. xanthus*. We deleted most of the *lexA* gene sequence, leaving only 53 upstream bases and 9 downstream bases (Fig. 1A), and the deletion mutant was named LA. The cell morphology of the mutant did not show filamentous cells like that of the *lexA* mutation in *Clostridium difficile* [21]. Using genomic DNA or the cDNA reverse-transcribed from the total RNA as templates, no complete *lexA* gene was amplified by PCR from the *lexA* mutant, except for a 70-bp fragment, which was confirmed by sequencing (Fig. 1A). The absence of *lexA* had little effect on the UV-C resistance of *M. xanthus* cells (Fig. 1B), but delayed the growth of this strain (Fig. 1C). Compared with the wild type, the *lexA* mutant showed a prolonged lag phase (from 24 to 36 h). UV-C radiation treatment further prolonged the lag phase of both the mutant and wild-type strains, which was attributed to DNA damage and cell death. It is noteworthy that the mutant shows more resistance to UV-C radiation at a low dosage (10 J/m^2^) than that of wild-type strain (Figs. 1B and 1C), which has been thought to be caused by the release of SOS response [4, 5].

In the sporulation process, both DK1622 and LA showed cell aggregation to form visible fruiting body at 24 h, and there was no significant difference in their fruiting body formation and sporulation. When the strains were UV-C irradiated, the aggregation abilities of both DK1622 and LA were delayed. With the increase of radiation dose, the visible fruiting bodies and the final number of spores were also decreased gradually and correspondingly with almost no difference in DK1622 and LA treated with the same dose of radiation (Figs. 1D and 1E).

According to the previous reports on other bacteria, the *lexA* deletion mutant has a distinct SOS-response phenotype, such as inhibited cell division and sporulation, increased UV-C resistance and mutation rate [21-24]. However, the *lexA* gene deletion in *M. xanthus* has no significant effect on bacterial morphology, UV-C resistance, and sporulation, but showed delayed growth.

### Transcriptional Profiles of UV-Induced *M. xanthus*

We inspected the SOS response by RNA sequencing in the UV-C irradiated *M. xanthus* with 15 J/m^2^, at which UV significantly affected the survival rates and growth curves of both wild type and mutant (Fig. 1). A total of 1,439 differentially expressed genes (DEGs) were identified in UV-irradiated *M. xanthus*, including 651 upregulated DEGs and 788 downregulated DEGs (Fig. S1A).

The UV-induced genes (651 upregulated DEGs) were gene-ontology (GO) enriched into 47 functional items (*p*-values < 0.05) (Fig. S1B), divided into three independent categories: biological process (BP) containing 26 items, accounting for 55.3% of the total items; cellular component (CC) containing 2 items (4.3%); molecular function (MF) containing 19 items (40.4%). There were 8 GO items with more significantly enriched genes (>30 DEGs), including 4 BP items (cellular component organization, cellular component organization or biogenesis, DNA metabolic process, and nucleic acid metabolic process) and 4 MF items (heterocyclic compound binding, organic cyclic compound binding, DNA binding, and nucleic acid binding). On the other hand, the 788 downregulated DEGs were enriched in 39 GO items of BP (19 items), CC (7 items) and MF (13 items) groups (Fig. S1C). The most enrichment in 8 GO items (>30 DEGs in each item), were membrane, transporter activity, intracellular membrane bound organelle, transmembrane transporter activity, localization, establishment of localization and oxidation-reduction process.

**Fig. 2:**
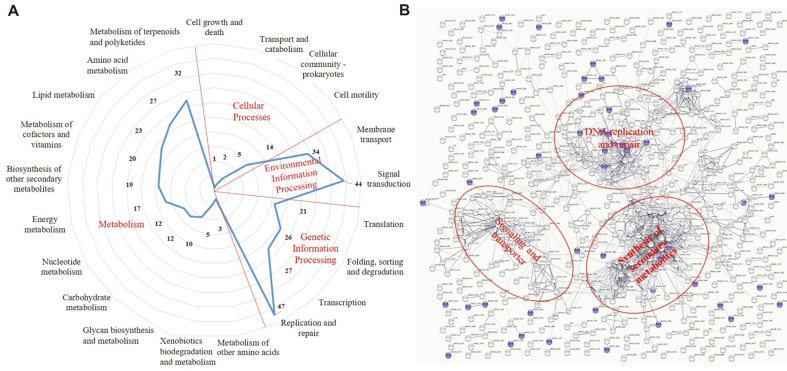
Functional classification of the UV-induced genes. (**A**) Gene distribution according to KEGG pathways. (**B**) Functional protein association networks using STRING. The DEGs affected by LA are shown in blue in B.

Furtherly, KEGG pathway enrichment analysis of the UV-induced genes led to identification of 21 statistically enriched pathways (Fig. 2A), distributed in metabolism, genetic information processing, environmental information processing, and cellular processes. Obviously, the UV-induced genes were enriched in three pathways of replication and repair, signal transduction, and metabolism of terpenoids and polyketides.

Bacterial SOS response is characterized by the induction of the genes related to DNA damage and repair [1,2,4]. In *M. xanthus*, the DNA replication and repair pathway had the largest number of the UV-induced genes (47 genes), including the typical SOS genes *lexA*, *recA*, *uvrA*, *ruvAB*, and *recN*. These DNA replication and repair genes could be further sub-classified to DNA replication, base excision repair, nucleic acid excision repair, mismatch repair, recombination repair, and DnaE2-catalyzed TLS processes (Table S1). However, compared with that in *E. coli* [25], the number of DNA replication and repair genes induced by UV-C radiation decreased in *M. xanthus*. Some known bacterial UV-induced genes were not induced in *M. xanthus*, such as *recF*, *recO*, *recR*, *rvrC*, *mutY*, *ftsK*, *ruvA*, and *ssb*.

The second pathway of gene enrichment was metabolism of terpenoids and polyketides, which is known to be related to secondary metabolites. Using antiSMASH [26], 802 genes involved in 23 secondary metabolite biosynthetic gene clusters (BGCs) were identified in *M. xanthus* DK1622, of which 217 genes were affected by UV-C radiation and distributed in all the 23 gene clusters, including 126 upregulated genes and 91 downregulated genes (Table S2). The 126 upregulated secondary metabolism related genes were mainly distributed in metabolisms of terpenoids and polyketides, amino acids and lipids, and metabolism of cofactors and vitamins, which enrich most of the UV-induced metabolic genes (Fig. 2A).

The third pathway of gene enrichment was signal transduction of environmental information processing. *M. xanthus* contains many genes involved in signal transduction pathways, including the signal pathways for SOS response [18]. The UV-induced 44 signal transduction genes and 34 membrane transport genes formed the enrichment region of environmental information processing. So many signal proteins are involved in the SOS response, which is helpful to understand the mechanism of LexA-independent SOS induction in *M. xanthus*.

Meanwhile, we used STRING to search for functional protein association networks of the UV-induced genes [27], and 27 clusters were significantly enriched (Table S3). These local network clusters can be divided into three functional enrichment regions, secondary metabolite, DNA replication and repair, signaling and transporter (Fig. 2B), which is consistent with the functional areas formed by the KEGG pathway enrichment in Fig. 2A.

### Transcriptional Profiles of the UV-Irradiated *lexA* Mutant

UV-C irradiation treatment on the *lexA* mutant (LA) resulted in 1,086 DEGs (Fig. S2A), including 412 upregulated DEGs and 674 downregulated DEGs. The induced genes included those conservative SOS genes (*recA*, *lexA*, *recN*, *uvrA*, and so on), which suggested that the absence of LexA does not affect the induction of SOS response.

Comparing the DEGs between LAUV vs LA and DKUV vs DK1622 (Fig. S2B), 376 DEGs were consistent, and 710 DEGs in LA were inconsistent with those in DK1622. In the upregulated 412 genes of UV-irradiated LA, 289 were synchronously upregulated in UV-irradiated DK1622, occupying more than 70% of the total upregulated 412 genes. In the downregulated DEGs, however, only less than 10% of DEGs (66/674) were consistent. Thus, the difference of DEGs between DK1622 and the LA mutant was mainly due to the downregulated DEGs.

GO analysis showed that the upregulated DEGs in the UV-irradiated LA strain were mainly involved in the acyl transfer groups, DNA damage and repair, similar to that of the wild-type strain. The downregulated DEGs were enriched into cellular glucan metabolic process, electron carrier activity, cellulose metabolic process, catabolic process and other metabolic processes (Table S4), which were obviously different from that of wild type. As a result, the difference of DEGs between UV-irradiated LA and wild-type strains might be due to the indirect effect of the inhibition by LexA on bacterial growth.

According to the KEGG pathways, the UV-induced genes in LA were also enriched in replication and repair, signal transduction, and metabolism of terpenoids and polyketides (Fig. S2C), which were similar to that in wild type. The above results showed that the deletion of *lexA* had less efficient effect on the expression of SOS-induced genes, but more on the bacterial growth, which is consistent with the mutant analysis (Fig. 1).

### LexA Repressor Acts on a Small Number of UV-Induced Genes

**Fig. 3:**
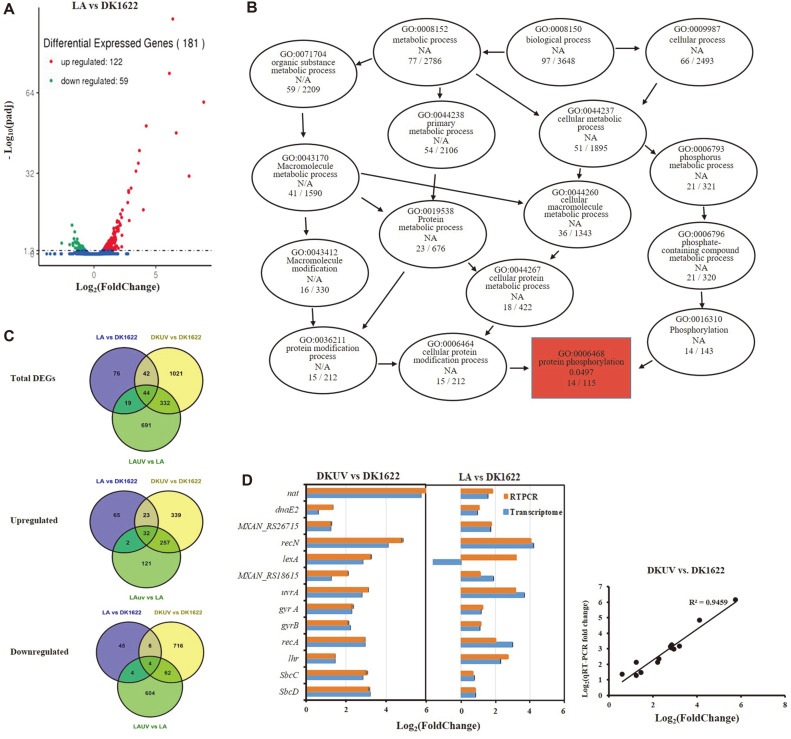
Analyses of the DEGs from *lexA* mutant (LA). (**A**) Volcano plot of DEGs in *lexA* mutant; (**B**) GO enrichment in LA; (**C**) Venn diagram comparison from the combination of LA vs DK1622 and DKUV vs DK1622. (**D**) RT-PCR verification of 13 differentially expressed DNA replication and repair genes.

Compared with the expression profile of wild-type strain, the absence of *lexA* resulted in 181 DEGs in *M. xanthus* (Fig. 3A, Table S5). Among them, 122 were upregulated and 59 were downregulated, accordant with the expectation of LexA as a repressor of SOS response. The DEGs affected by LexA were enriched into 52 GO items, but only the protein phosphorylation exhibited significant difference (*p* < 0.05) (Fig. 3B). Protein phosphorylation is the main mechanism of signal transduction that enables bacteria to rapidly respond to environmental changes by controlling the functional properties of proteins in response to external stimuli [28-30]. Notably, the DEGs affected by LexA were not obviously enriched in any KEGG pathway or clusters in functional protein association networks (Fig. 2B).

Comparing the DEGs between LA vs DK1622 and DKUV vs DK1622, 86 DEGs (approximately 47.5% of the total 181 DEGs in LA) were consistent, which suggested that these genes were involved in UV-induced SOS response (Fig. 3C). In the upregulated DEGs of these two groups, 55 of 122 up regulated DEGs (45%) in LA were induced by UV-C in wild type. Meanwhile, 10 of 59 down regulated DEGs (16.9%) in LA were inhibited in UV-irradiated wild-type strain. Thus, LexA might act as an inhibitor in the UV-induced *M. xanthus* SOS response.

In a canonical SOS response, LexA inhibited DNA replication and repair. Thirteen DEGs in the *lexA* mutant belonged to the DNA replication and repair pathway, of which 12 were UV-upregulated, except *lexA*. RT-PCR analysis also confirmed the transcriptional change of the 13 genes in *lexA* mutant (Fig. 3D). The *lexA* gene was also upregulated in RT-PCR inspection, but downregulated in LA transcriptome, possibly due to the different experimental methods. In transcriptome analysis, the transcriptional sequences of genes are detected and counted. Because most of the coding sequences of *lexA* gene were missing in the mutant and only the sequences remained at the C- and N- terminal of *lexA* gene could be detected by transcriptome analysis, which showed downregulated expression in the transcriptome of LA. In RT-PCR analysis, the N-terminal retained sequences of *lexA* gene were used as the detection target which has no difference between wild type and mutant, and the result showed that LexA also repressed its own expression (Fig. 3D).

However, it was true that LexA only affected a fraction of the UV-induced genes, which were scattered and had no obvious function enrichment (marked in blue in Fig. 2B). There were 47 replication and repair genes that were induced by UV-C, and only 13 of them were repressed by LexA and the expression of the other 34 genes was LexA independent (Table S1). Similarly, induction of secondary metabolite synthesis was a characteristic of the UV-induced SOS response in *M. xanthus*, and LexA affected the expression of secondary metabolism genes, but the number of DEGs was significantly less than that induced by UV-C. Compared with the above 217 secondary metabolite synthesis genes affected by UV-C, the deletion of *lexA* only affected 20 of them (Table S2).

In sum, LexA affects the expression of SOS-induced genes involved in DNA replication and repair and metabolism, but the proportion of SOS genes regulated by LexA is very small among the total of SOS genes.

### LexA Directly Inhibited Only Five Regulatory Genes Including Itself

Comparing the upregulated DEGs between LA vs DK1622 and DKUV vs DK1622 (Fig. 3C), 55 SOS genes (not including *lexA*) were repressed by LexA, of which 32 could be further upregulated by UV-C in the LA, suggesting that these genes had other regulatory pathways in addition to the LexA-dependent regulation. The other 23 DEGs were not UV-induced in the LA mutant, which probably suggested that they might be affected by LexA only. Interestingly, these 23 genes contained the known classical LexA regulatory genes including *lexA*, *recN*, *uvrA*, *dnaE2*, and *recA2*.

**Fig. 4:**
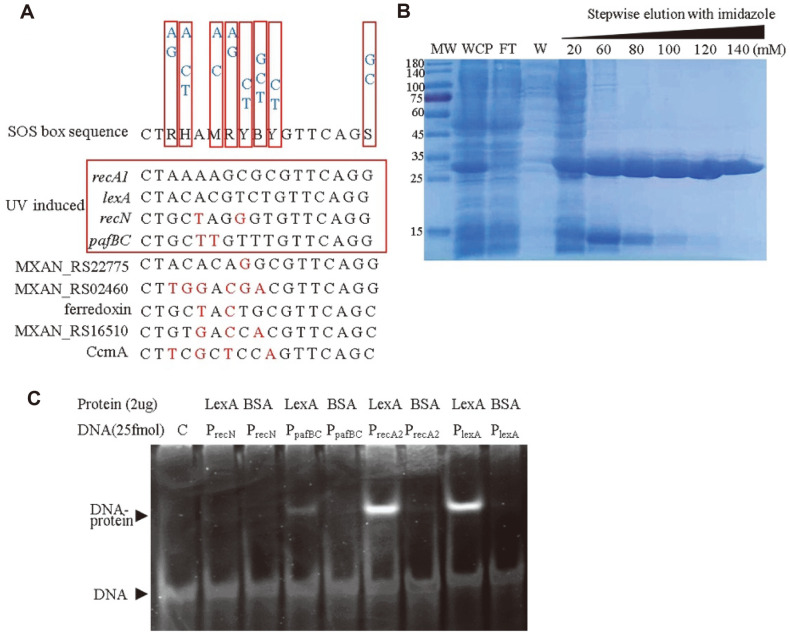
DNA binding sequences of LexA. (**A**) Conserved SOS box of LexA regulatory genes. (**B**) The expression and purification of LexA. (**C**) Gel shift analysis of LexA binding to the SOS boxes of *recN*, *recA2*, *lexA* and *pafB*C.

The binding pattern of *M. xanthus* SOS box (CTRHAMRYBYGTTCAGS) is an imperfect palindrome sequence with the necessary internal bases for LexA binding [18]. We compared the core promoter regions of the 56 LexA-repressed DEGs (including *lexA* gene), and found that only *recA2* and *lexA* had the fully matched SOS box sequences in their core promoter regions (Fig. 4A). The promoter of *recN* has a similar SOS box region with two different bases (CTGCTAGGGTGTTCAGG) from the reported SOS box. In addition, the promoter of *pafB*C operon, which contains the genes *pafB* (MXAN_RS06580), *pafC* (MXAN_RS06585) and *cyaA* (adenylate cyclase, MXAN_RS06590), also has a similar SOS box (CTGCTTGTTTGTTCAGG).

We expressed and purified the LexA protein (Fig. 4B) and analyzed its binding ability to the four SOS box sequences by gel shift analysis (Fig. 4C). The result showed that that LexA protein could bind the SOS boxes of *lexA* and *recA2* obviously, bind the SOS box of *pafBC* weakly, and could not bind the SOS box of *recN* gene. Furthermore, we searched the whole genome of *M. xanthus* for the SOS box sequence and found five other genes with similar sequences in their promoters, including ferredoxin, heme ABC exporter CcmA and three function-unknown genes (Fig. 4A).

Although LexA affected 56 SOS genes, only the expression of *recA2*, *lexA*, *pafB*, *pafC*, and *cyaA* was directly repressed by LexA. The expression of the other 51 genes was indirectly regulated by LexA. In our previous work, we found that RecA2 was not involved in the DNA recombination and LexA autocleavage, but, instead, in the regulation on cell growth and antioxidant [20]. Compared with the reported transcriptome data of *recA2* mutant, 12 of the 51 genes were expressed in a RecA2-dependent manner (as shown in Fig. S3). Similarly, PafBC encoded by *pafB* and *pafC* was involved in a LexA/RecA-independent DNA damage response [31], and 24 of the 51 genes had the PafBC-binding sequence in their promoters. The adenylyl cyclase encoded by gene *cyaA* catalyzed the synthesis of cyclic AMP (cAMP), which is one of the most common signaling molecules and is widely distributed from prokaryotes to eukaryotes. Thus, we concluded that LexA has shrunk its regulation on the SOS genes, and the SOS response is more dependent on other signaling pathways. In *M. xanthus*, LexA does not directly control DNA damage repair related genes, but controls five transcription regulatory protein genes (*recA2*, *lexA*, *pafB*, *pafC*, and *cyaA*) to affect the SOS gene expression depending on other signaling pathways (Fig. S3).

## Conclusion

This work is part of the research on the replication, recombination and repair in myxobacteria. UV-C irradiation affected the expression of a large number of genes in *M. xanthus*, of which 651 genes were up regulated and were mainly distributed in DNA replication and repair, signal transduction, and secondary metabolite synthesis.

The absence of *lexA* gene in *M. xanthus* did not show the *lexA* deletion phenotype common in other bacteria, except for delayed growth. Transcriptome analysis found that the regulatory range of LexA was significantly shrinking in *M. xanthus*, and the absence of LexA only enhanced the expression of 56 SOS-inducible genes, which was significantly less than the number of SOS genes affected by LexA in *E. coli* [25]. The 56 genes contained a small part of SOS-induced genes involved in DNA replication and repair and metabolism, but no genes related to sporulation and cell division of *Myxococcus*. Further LexA binding analysis found that only five SOS-induced genes (recA2, *lexA*, *pafB*, *pafC*, and *cyaA*) were directly repressed by LexA.

## Supplemental Materials

Supplementary data for this paper are available on-line only at http://jmb.or.kr.
