# Supplementary material for: The Regulation of LexA on UV-Induced SOS Response in Myxococcus xanthus Based on Transcriptome Analysis
Source: J Microbiol Biotechnol. 2021 May 24;31(7):912–20. doi: 10.4014/jmb.2103.03047 (PMC9705874; doi:10.4014/jmb.2103.03047)
Supplement: Supplementary file 1 [file jmb-31-7-912-supple.pdf]

Supplementary materials

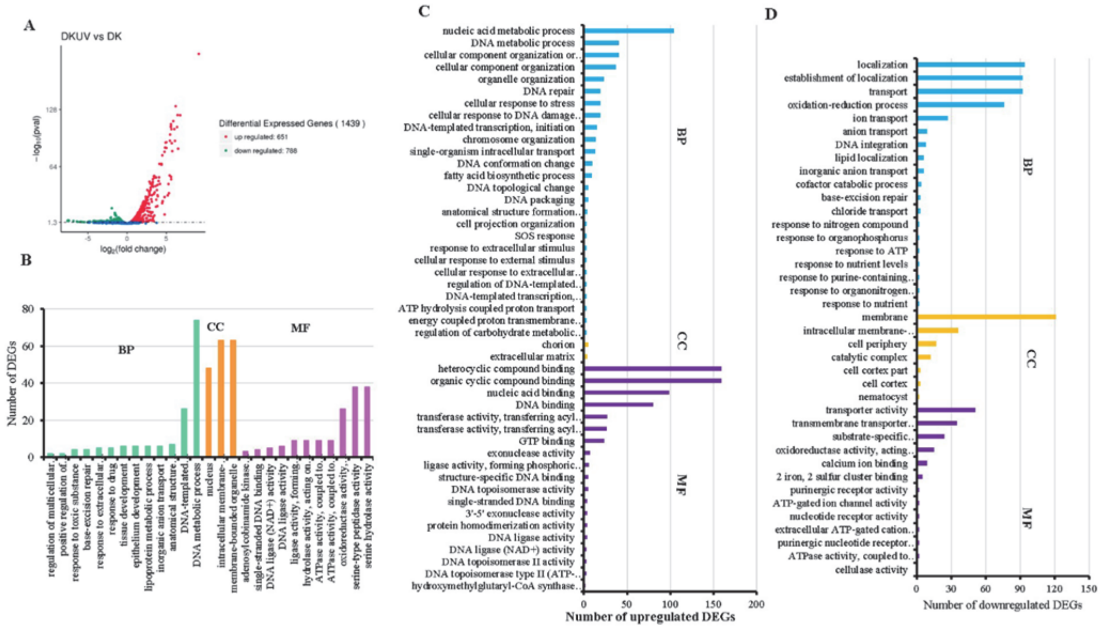

Figure S1. Analysis of differentially expressed genes (DEGs) in UV-C irradiated *M. xanthus*. (A) Volcano plot of DEGs; (B) GO analysis of upregulated DEGs (C) GO analysis of downregulated DEGs.

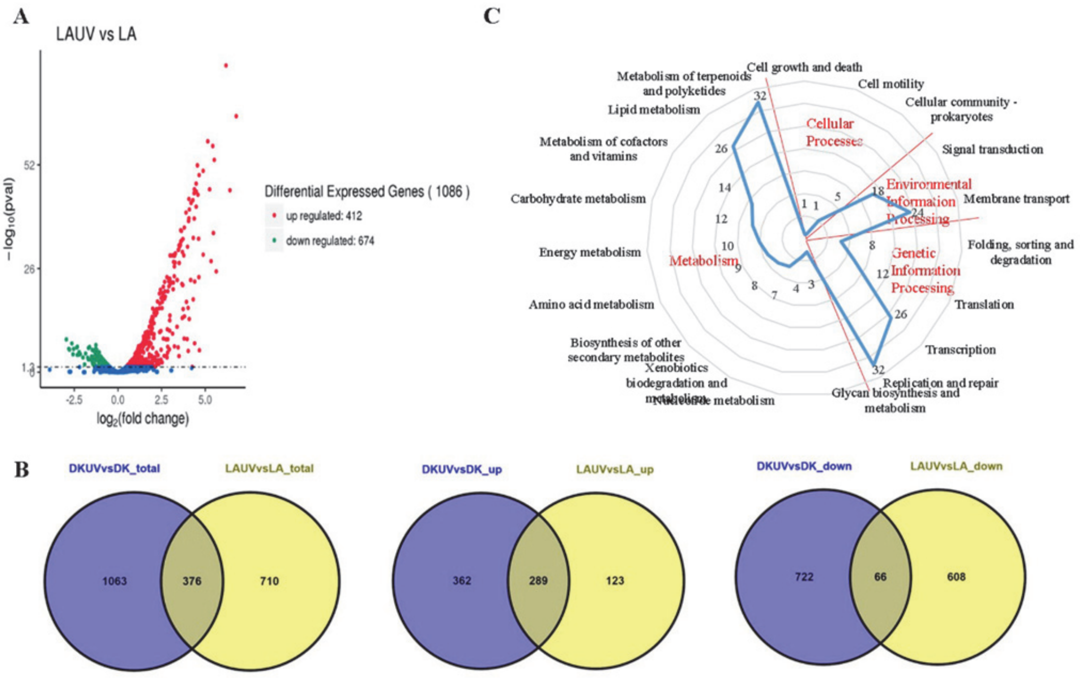

Figure S2. Analysis of differentially expressed genes (DEGs) in UV-C irradiated LA strain. (A) Volcano plot of DEGs; (B) KEGG pathways enrichment of upregulated DEGs (C) Venn diagram comparison of the DEGs from the combinations LAUV vs LA and DKUV vs DK1622.

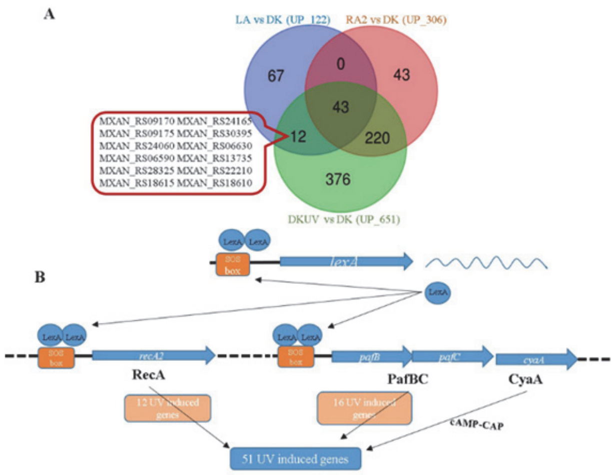

Figure S3. Predicted SOS regulation in *M. xanthus.* (A) Venn diagram of upregulated genes in LA vs DK1622, DKUV vs DK1622 and RA2 vs DK1622. (B) Genes regulated by LexA in *M. xanthus.*

A

Sample name	Raw reads	Clean reads	clean bases	Error rate(%)	Q20(%)	Q30(%)	GC content(%)
**DK1**	18897054	18377100	2.76G	0.02	98.31	95.03	67.82
**DK2**	21908944	21541630	3.23G	0.02	98.19	94.6	67.46
**DK3**	15957614	15678290	2.35G	0.03	97.75	93.52	67.63
**DK_UV1**	19954130	19569536	2.94G	0.02	98.14	94.51	67.72
**DK_UV2**	20658672	20324744	3.05G	0.02	98.13	94.45	67.58
**DK_UV3**	22708818	22348186	3.35G	0.03	97.83	93.88	67.48
**LA1**	19896734	19529714	2.93G	0.02	98.08	94.37	67.66
**LA2**	14322054	14073360	2.11G	0.02	98.25	94.75	67.96
**LA3**	22451474	21880532	3.28G	0.03	98	94.17	67.41
**LA_UV1**	19636918	19335366	2.9G	0.02	98.2	94.63	67.58
**LA_UV2**	18817980	18465436	2.77G	0.02	98.26	94.82	68.16
**LA_UV3**	18867550	18479130	2.77G	0.02	98.17	94.56	67.55

B

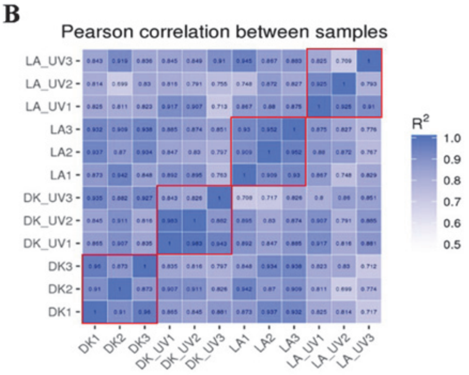

Figure S4. RNA sequencing. (A) Sequencing quality information list. (B) Heatmap of sample correlation analysis result base on global transcriptome expression of wild-type strain and *lexA* mutant under radiation stress.

Table S1. DNA replication and repair genes induced by UV-C radiation in *M. xanthus*. The upregulated genes in *lexA* mutant were marked in red.

GeneID	Protein	DKUV vs. DK1622		LA VS. DK1622	
		log2FoldChange	pval	log2FoldChange	pval
MXAN_RS25965	RecN	4.119444352	9.33E-68	4.221505731	1.55E-51
MXAN_RS12615	UvrA	2.79965714	1.72E-36	3.674296879	9.36E-42
MXAN_RS06730	RecA2	2.963576507	4.07E-38	3.00030397	1.11E-29
MXAN_RS04795	Helicase, Lhr	1.463354738	2.57E-12	2.296686342	7.60E-21
MXAN_RS18615	helicase, YprA	1.256079907	1.97E-10	1.881462008	7.78E-14
MXAN_RS21600	LexA	2.856656967	1.33E-35	-1.773867382	3.99E-12
MXAN_RS26715	DEAD/DEAH box helicase	1.24125619	1.31E-05	1.729931049	2.71E-07
MXAN_RS33805	N-acetyltransferase	5.761293942	4.27E-82	1.558372114	4.54E-07
MXAN_RS12020	GyrA	2.281379899	3.38E-25	1.157047046	1.53E-06
MXAN_RS12015	GyrB	2.215821756	1.05E-23	1.088699534	1.14E-05

MXAN_RS04610	DNA repair protein SbcD	3.212974538	3.95E-46	0.838663196	0.000103068
MXAN_RS04615	DNA repair protein SbcC	2.841598412	2.26E-34	0.766988316	0.000609849
MXAN_RS28325	DNA polymerase III subunit alpha	0.613967862	0.007462047	0.941606852	0.011892181
MXAN_RS19360	error-prone DNA polymerase	3.062955407	3.73E-12	1.32964464	0.055980745
MXAN_RS06060	RadA	1.684544303	8.31E-15	0.501959707	0.061326435
MXAN_RS02985	ATP-dependent DNA ligase	1.806105994	6.23E-20	0.343975514	0.11876033
MXAN_RS01295	DNA gyrase subunit B	0.459389481	0.044410506	0.337338863	0.166164173
MXAN_RS15255	endonuclease III	2.109229555	1.00E-09	1.159336293	0.289598048
MXAN_RS02595	RecQ	1.141533148	3.15E-06	0.236354618	0.330048671
MXAN_RS06925	primosomal protein N'	2.491099947	2.27E-29	0.197724801	0.378264203
MXAN_RS05180	N-acetyltransferase	1.34466735	3.89E-07	-0.384036082	0.396291155

MXAN_RS07590	DNA starvation/stationary phase protection protein	0.770071369	0.002404722	0.18364224	0.474730046
MXAN_RS19405	ImuA	4.492607537	2.24E-77	0.722249951	0.491657694
MXAN_RS07215	HNH endonuclease	3.880637615	1.46E-32	-0.231946588	0.781503764
MXAN_RS12155	ParB	1.616486273	1.11E-15	0.538941257	0.820884176
MXAN_RS12305	DNA polymerase IV	3.879824263	4.45E-44	0.538941257	0.820884176
MXAN_RS05140	SSB	1.461331551	3.12E-10	0.040628337	0.88160119
MXAN_RS07875	DNA polymerase II	3.151000946	3.57E-25	-0.045799175	0.889249238
MXAN_RS05970	N-acetyltransferase	1.848301069	3.53E-06	0.089285034	0.901847011
MXAN_RS27320	DNA ligase, LigA	1.276577359	4.99E-09	0.426884262	0.970057287
MXAN_RS13900	endonuclease	1.647383189	1.74E-12	-0.209565972	1
MXAN_RS16220	HNH endonuclease	0.760521765	0.004972791	-0.01781213	1

MXAN_RS16500	DNA helicase RecG	0.86923212	3.11E-05	-0.086465316	1
MXAN_RS16965	integration factor subunit beta	0.813944208	0.024378043	0.081926863	1
MXAN_RS19045	ParA	1.06891837	4.40E-08	0.153228614	1
MXAN_RS19400	ImuB	3.917312763	3.09E-31	0.260756111	1
MXAN_RS20345	mismatch repair protein, MutS	0.483114243	0.031972366	0.075062099	1
MXAN_RS20870	FAD-binding protein	1.317746718	3.20E-09	-0.123875301	1
MXAN_RS21420	JmjC protein	0.758372595	0.000202967	-0.193683131	1
MXAN_RS21430	JmjC protein	0.793972884	0.000614377	-0.462492789	1
MXAN_RS23655	DNA replication protein	0.75149075	0.000721255	0.118590888	1
MXAN_RS23795	chromosome protein SMC	1.514053317	3.80E-11	0.352313448	1
MXAN_RS24700	replicative DNA helicase	2.245310309	2.10E-23	0.216006311	1
MXAN_RS29975	N-acetyltransferase	0.423477358	0.048044889	-0.297881133	1

MXAN_RS30350	DNA polymerase	3.292053004	1.60E-50	-0.069889679	1
MXAN_RS32480	regulatory protein RecX	1.753304464	2.88E-16	0.590125927	1
MXAN_RS36950	DNA-binding protein	1.084277261	0.001986127	0.257402548	1

Table S2. DEGs related to secondary metabolite.

Regions	Type	From	To	Gene coverage	Gene number	DEGs after UV irradiation			DEGs affected by LexA		
						Total	Upregulated	Downregulated	Total	Upregulated	Downregulated
Region 1	terpene	1,015,682	1,032,939	MXAN_RS04270-MXAN_RS04345	16	2	0	2	0	0	0
Region 2	betalactone, NRPS	1,490,426	1,537,319	MXAN_RS06165-MXAN_RS06345	38	14	13	1	3	0	3
Region 3	NRPS-like	1,771,438	1,813,469	MXAN_RS07325-MXAN_RS07490	35	6	4	2	0	0	0
Region 4	NRPS	1,863,446	1,927,180	MXAN_RS07720-MXAN_RS07895	36	5	1	4	1	1	0
Region 5	TfuA-related	2,778,046	2,800,369	MXAN_RS11555-M	18	1	0	1	1	0	1

				XAN_RS11640							
Region 6	NRPS-like, T1PKS	3,235,611	3,284,053	MXAN_RS13480 -MXAN_RS13635	31	8	7	1	2	2	0
Region 7	lanthipeptide	3,318,246	3,340,534	MXAN_RS13795-MXAN_RS13865	15	2	2	0	0	0	0
Region 8	T1PKS	4,013,494	4,058,514	MXAN_RS16715-MXAN_RS16860	31	7	4	3	4	3	1
Region 9	bacteriocin	4,135,538	4,145,774	MXAN_RS17220-MXAN_RS17255	8	2	2	0	0	0	0
Region 10	NRPS, T1PKS	4,202,141	4,368,221	MXAN_RS36820-MXAN_RS17745	56	25	1	24	0	0	0
Region 11	NRPS, T1PKS	4,481,549	4,563,638	MXAN_RS18260 -MXAN_RS18415	32	3	3	0	0	0	0

Region 12	transAT-PKS, NRPS, PKS-like	4,710,911	4,822,556	MXAN_RS19030-MXAN_RS19240	43	19	16	3	1	0	1
Region 13	NRPS, T1PKS	4,855,909	4,923,967	MXAN_RS19380-MXAN_RS19540	33	13	6	7	0	0	0
Region 14	T1PKS, NRPS	4,977,639	5,044,797	MXAN_RS36870-MXAN_RS19925	41	7	6	1	0	0	0
Region 15	bacteriocin	5,105,157	5,116,326	MXAN_RS19965-MXAN_RS20220	57	7	2	5	0	0	0
Region 16	T1PKS, NRPS	5,235,101	5,320,830	MXAN_RS20735-MXAN_RS20970	48	24	17	7	1	0	1
Region 17	NRPS, T1PKS	5,387,898	5,488,693	MXAN_RS21280-MXAN_RS21555	56	15	8	7	0	0	0
Region 18	NRPS, T1PKS	5,585,364	5,672,405	MXAN_RS21890-M	36	13	9	4	0	0	0

				XAN_RS22060							
Region 19	NRPS, bacteriocin	5,715,538	5,794,912	MXAN_RS22215-MXAN_RS22415	41	10	3	7	3	0	3
Region 20	thiopeptide, bacteriocin	6,157,224	6,205,340	MXAN_RS23895-MXAN_RS24085	38	13	5	8	3	3	0
Region 21	terpene	7,706,986	7,729,253	MXAN_RS30245-MXAN_RS30320	16	2	0	2	0	0	0
Region 22	ladderane, lanthipeptide	7,855,962	7,897,173	MXAN_RS30880-MXAN_RS31045	35	17	15	2	0	0	0
Region 23	T3PKS	8,147,550	8,188,632	MXAN_RS32035-MXAN_RS32245	42	2	2	0	1	0	1
Total					802	217	126	91		9	11

Table S3. local network cluster (STRING) of UV induced genes

**cluster**	**description**	**count in network**	**strength**	**false discovery rate**
CL:7423	This enzymatic domain is part of bacterial polyketide synthases and catalyses the first step in the reductive modification of the beta-carbonyl	6 of 6	1.07	0.0041
CL:7579	FabA-like domain, and Biotin metabolism	5 of 5	1.07	0.0141
CL:3552	mixed, incl. DTXR-type HTH domain, and Outer membrane lipoprotein-sorting protein	5 of 5	1.07	0.0141
CL:3549	mostly uncharacterized, incl. DTXR-type HTH domain, and Outer membrane lipoprotein-sorting protein	11 of 14	0.97	5.24E-05
CL:7339	Polyketide synthase, phosphopantetheine-binding domain, and Hydroxymethylglutaryl-coenzyme A synthase N terminal	29 of 50	0.83	7.31E-11
CL:7558	Fatty acid biosynthesis, and Metallo-beta-lactamase domain-containing protein	9 of 17	0.79	0.0032

CL:7387	mixed, incl. Polyketide synthase, ketoreductase domain, and Hydroxymethylglutaryl-coenzyme A synthase N terminal	9 of 17	0.79	0.0032
CL:11769	mixed, incl. Type VI secretion system, VipAand Bacterial secretion system	10 of 20	0.77	0.002
CL:7340	Phosphopantetheine attachment site, and ACP-like superfamily	20 of 41	0.76	1.14E-06
CL:7341	Phosphopantetheine attachment site, and Thioester reductase-like domain	11 of 23	0.75	1.30E-03
CL:7554	mixed, incl. Fatty acid biosynthesis, and Acyl carrier protein (ACP)	18 of 39	0.73	1.05E-05
CL:7337	ACP-like superfamily, and Biosynthesis of siderophore group nonribosomal peptides	30 of 68	0.72	4.06E-09
CL:7343	Phosphopantetheine attachment site, and Thioester reductase-like domain	8 of 18	0.72	0.0146
CL:5697	mixed, incl. Excision nuclease, and impB/mucB/samB family C-terminal domain	7 of 16	0.71	0.0336
CL:7336	mixed, incl. ACP-like superfamily, and Biosynthesis of siderophore group nonribosomal peptides	31 of 73	0.7	4.06E-09
CL:3487	mixed, incl. Flagellar assembly, and Bacterial secretion system	19 of 45	0.7	1.33E-05
CL:7556	Fatty acid biosynthesis, and Malonyl CoA-acyl carrier protein transacylase	12 of 28	0.7	0.0013
CL:4103	mostly uncharacterized, incl. Small GTPase, and NACHT domain	7 of 17	0.69	4.30E-02

CL:11767	mixed, incl. Domain of unknown function DUF2169, and Domain of unknown function DUF4150	11 of 27	0.68	0.0033
CL:7328	mixed, incl. ACP-like superfamily, and Fatty acid biosynthesis	66 of 178	0.64	2.98E-17
CL:7329	mixed, incl. ACP-like superfamily, and Fatty acid biosynthesis	63 of 169	0.64	6.17E-17
CL:7333	mixed, incl. ACP-like superfamily, and Biosynthesis of siderophore group nonribosomal peptides	34 of 91	0.64	4.95E-09
CL:11766	mixed, incl. Domain of unknown function DUF2169, and Domain of unknown function DUF4150	12 of 34	0.62	0.0045
CL:7331	mixed, incl. ACP-like superfamily, and Biosynthesis of siderophore group nonribosomal peptides	37 of 106	0.61	4.06E-09
CL:11764	mixed, incl. Domain of unknown function DUF2169, and Domain of unknown function DUF4150	15 of 46	0.58	0.0019
CL:5691	mixed, incl. DNA repair, and Mismatch repair	19 of 65	0.54	0.00084
CL:5693	DNA repair, and Mismatch repair	13 of 44	0.54	0.01

Table S4. GO enrichment of DEGs in UV-irradiated *lexA* mutant

GO_accession	Description	Term_type	Corrected_pValue	DEG_item	Bg_item	Gene_names
**GO enrichment of upregulated DEGs in UV-irradiated *lexA* mutant**						
GO:0016747	transferase activity, transferring acyl groups other than amino-acyl groups	molecular_function	0.0099306	20	132	MXAN_RS19130, MXAN_RS33805, MXAN_RS19175, MXAN_RS05180, MXAN_RS19825, MXAN_RS30975, MXAN_RS35585, MXAN_RS20845, MXAN_RS31000, MXAN_RS20860, MXAN_RS05970, MXAN_RS20855, MXAN_RS19140, MXAN_RS30995, MXAN_RS16780, MXAN_RS20850, MXAN_RS30970, MXAN_RS20820, MXAN_RS19190, MXAN_RS20865

GO:0016746	transferase activity, transferring acyl groups	molecular_function	0.013815	21	163	MXAN_RS20855, MXAN_RS16780, MXAN_RS19140, MXAN_RS30995, MXAN_RS30970, MXAN_RS20850, MXAN_RS20865, MXAN_RS20820, MXAN_RS19190, MXAN_RS31000, MXAN_RS20860, MXAN_RS05970, MXAN_RS30975, MXAN_RS19825, MXAN_RS35585, MXAN_RS20845, MXAN_RS33805, MXAN_RS19130, MXAN_RS35570, MXAN_RS19175, MXAN_RS05180
GO:0006281	DNA repair	biological_process	0.049761	13	110	MXAN_RS02985, MXAN_RS23445, MXAN_RS23440, MXAN_RS15255, MXAN_RS18335, MXAN_RS06060,

						MXAN_RS24185, MXAN_RS32480, MXAN_RS35785, MXAN_RS19405, MXAN_RS19400, MXAN_RS19835, MXAN_RS12305
GO:0003684	damaged DNA binding	molecular_function	0.049761	3	7	MXAN_RS19400, MXAN_RS24185, MXAN_RS12305
GO:0006974	cellular response to DNA damage stimulus	biological_process	0.049761	13	117	MXAN_RS19405, MXAN_RS19400, MXAN_RS32480, MXAN_RS24185, MXAN_RS35785, MXAN_RS12305, MXAN_RS19835, MXAN_RS15255, MXAN_RS18335, MXAN_RS02985, MXAN_RS23445, MXAN_RS23440, MXAN_RS06060
GO:0033554	cellular response to stress	biological_process	0.049761	13	118	MXAN_RS35785, MXAN_RS24185, MXAN_RS32480, MXAN_RS19405, MXAN_RS19400, MXAN_RS19835,

						MXAN_RS12305, MXAN_RS23445, MXAN_RS02985, MXAN_RS23440, MXAN_RS15255, MXAN_RS18335, MXAN_RS06060
GO:0008452	RNA ligase activity	molecular_function	0.049761	3	7	MXAN_RS07390, MXAN_RS00335, MXAN_RS05975
GO:0006259	DNA metabolic process	biological_process	0.049761	23	303	MXAN_RS19400, MXAN_RS10205, MXAN_RS19405, MXAN_RS01020, MXAN_RS02595, MXAN_RS24700, MXAN_RS32480, MXAN_RS24185, MXAN_RS12305, MXAN_RS19835, MXAN_RS15255, MXAN_RS18335, MXAN_RS23445, MXAN_RS02985, MXAN_RS06060, MXAN_RS12020, MXAN_RS35785, MXAN_RS01730,

						MXAN_RS15000, MXAN_RS37160, MXAN_RS23440, MXAN_RS19360, MXAN_RS33800
**GO enrichment of downregulated DEGs in UV-irradiated *lexA* mutant**						
GO:0006073	cellular glucan metabolic process	biological_process	0.014939	3	5	MXAN_RS17860, MXAN_RS23500, MXAN_RS27820
GO:0044042	glucan metabolic process	biological_process	0.014939	3	5	MXAN_RS27820, MXAN_RS23500, MXAN_RS17860
GO:0009055	electron carrier activity	molecular_function	0.024891	8	79	MXAN_RS34435, MXAN_RS30175, MXAN_RS26945, MXAN_RS10660, MXAN_RS29505, MXAN_RS26935, MXAN_RS04695, MXAN_RS20585
GO:0030243	cellulose metabolic process	biological_process	0.037393	2	2	MXAN_RS27820, MXAN_RS23500
GO:0020037	heme binding	molecular_function	0.039338	7	70	MXAN_RS10660, MXAN_RS26945, MXAN_RS34435, MXAN_RS29505,

						MXAN_RS26935, MXAN_RS04695, MXAN_RS30175
GO:0044264	cellular polysaccharide metabolic process	biological_process	0.039338	3	9	MXAN_RS17860, MXAN_RS23500, MXAN_RS27820
GO:0005976	polysaccharide metabolic process	biological_process	0.044262	4	22	MXAN_RS27820, MXAN_RS03990, MXAN_RS17860, MXAN_RS23500
GO:0055114	oxidation-reduction process	biological_process	0.044262	23	562	MXAN_RS2448, MXAN_RS33870, MXAN_RS14375, MXAN_RS15365, MXAN_RS10660, MXAN_RS28420, MXAN_RS30180, MXAN_RS18435, MXAN_RS01320, MXAN_RS28565, MXAN_RS35750, MXAN_RS34695, MXAN_RS18740, MXAN_RS20585, MXAN_RS33700, MXAN_RS26945, MXAN_RS04695,

						MXAN_RS21315, MXAN_RS23070, MXAN_RS25590, MXAN_RS19280, MXAN_RS34435, MXAN_RS17125
GO:0051273	beta-glucan metabolic process	biological_process	0.044262	2	3	MXAN_RS27820, MXAN_RS23500
GO:0046527	glucosyltransferase activity	molecular_function	0.044262	2	3	MXAN_RS27820, MXAN_RS17860
GO:0009056	catabolic process	biological_process	0.046409	9	130	MXAN_RS18270, MXAN_RS26860, MXAN_RS03990, MXAN_RS34070, MXAN_RS23070, MXAN_RS32610, MXAN_RS21995, MXAN_RS23500, MXAN_RS02780

Table S5. 181 DEGs in *lexA* mutant.

GeneID			Readcount_LA	Readcount_DK1622	log2FoldChange	pval
MXAN_RS00575	MXAN_0115	hypothetical protein	60.09526	131.9279	-0.98891	8.62E-06
MXAN_RS00655	MXAN_0133	hypothetical protein	209.0701	493.8597	-1.09087	1.12E-05
MXAN_RS03355	MXAN_0694	hypothetical protein	75.63211	501.9033	-2.61081	4.51E-07
MXAN_RS03675	MXAN_0762	hypothetical protein	36.41216	70.21213	-0.81293	0.000526
MXAN_RS03780	MXAN_0785	RNA polymerase subunit sigma-32	23.06237	73.05063	-1.50861	7.45E-05
MXAN_RS03875	MXAN_0805	peptidase M10	146.1098	497.7825	-1.61771	5.93E-09
MXAN_RS06230	MXAN_1289	hypothetical protein	37.90585	77.79793	-0.90816	0.000904
MXAN_RS06240	MXAN_1291	non-ribosomal peptide synthetase	118.8559	256.9041	-0.98311	0.000373
MXAN_RS06245	MXAN_1292	hypothetical protein	37.18805	84.8999	-1.06443	0.000255
MXAN_RS08095	MXAN_1668	hypothetical protein	82.89904	188.1217	-1.0554	7.11E-05

MXAN_RS08460	MXAN_1743	cytochrome P450	56.25764	107.0452	-0.78396	0.000223
MXAN_RS08465	MXAN_1744	lipoxygenase	107.8544	202.4229	-0.76603	0.00039
MXAN_RS11600	MXAN_2399	serine/threonine protein kinase	155.7276	312.6091	-0.87441	0.000256
MXAN_RS13375	MXAN_2760	peptidase M28	90.79762	165.7862	-0.72518	0.000879
MXAN_RS13755	MXAN_2840	serine/threonine protein kinase	48.09637	155.5776	-1.55451	8.61E-12
MXAN_RS14160	MXAN_2923	gliding motility protein	40.81234	93.90876	-1.06982	0.000107
MXAN_RS15385	MXAN_3174	hypothetical protein	24.8623	70.26204	-1.34243	9.81E-05
MXAN_RS16860	MXAN_3478	membrane protein	19.57243	54.06176	-1.3255	0.000333
MXAN_RS17975	MXAN_3706	thiol oxidoreductase	73.47168	141.0716	-0.78945	0.001027
MXAN_RS19215	MXAN_3953	hypothetical protein	99.93311	230.6159	-1.05741	2.13E-05
MXAN_RS20725	MXAN_4269	hypothetical protein	51.76866	101.7556	-0.84487	0.000734
MXAN_RS21600	MXAN_4446	LexA repressor	48.75428	184.5311	-1.77387	1.25E-14

MXAN_RS22270	MXAN_4589	PEGA domain-containing protein	53.41914	110.3904	-0.90278	1.34E-05
MXAN_RS22275	MXAN_4590	hypothetical protein	19.99237	45.91703	-1.05661	0.000974
MXAN_RS22280	MXAN_4591	serine/threonine protein kinase	33.49085	66.99087	-0.86014	0.000266
MXAN_RS24160	MXAN_4975	response regulator	34.01223	65.56362	-0.8004	0.000777
MXAN_RS24790	MXAN_5102	hypothetical protein	45.28914	127.4574	-1.35411	8.42E-09
MXAN_RS24815	MXAN_5107	argininosuccinate lyase	81.66013	162.3476	-0.87391	0.001101
MXAN_RS24820	MXAN_5108	argininosuccinate synthase	107.4885	206.3262	-0.81839	0.001171
MXAN_RS25000	MXAN_5146	hypothetical protein	45.2456	85.25576	-0.77134	0.000497
MXAN_RS26010	MXAN_5360	glycosyl hydrolase	73.32261	157.8593	-0.9562	3.33E-05
MXAN_RS26045	MXAN_5367	glycosyl transferase family 2	41.20546	84.88754	-0.90363	5.04E-05
MXAN_RS26595	MXAN_5485	hypothetical protein	1350.71	3429.142	-1.21699	1.50E-05
MXAN_RS27420	MXAN_5658	hypothetical protein	40.4194	89.85482	-1.02486	7.18E-05

MXAN_RS28375	MXAN_5853	sigma-54 dependent DNA-binding response regulator	107.3519	451.2101	-1.96366	2.23E-06
MXAN_RS29545	MXAN_6090	hypothetical protein	165.9492	422.363	-1.1987	4.20E-06
MXAN_RS29620	MXAN_6107	NADPH:quinone oxidoreductase	56.03631	111.2388	-0.84052	0.000263
MXAN_RS29745	MXAN_6134	Ig-like domain/kelch domain-containing protein	60.89132	134.2549	-0.99404	3.86E-05
MXAN_RS30150	MXAN_6219	hypothetical protein	31.70157	77.08468	-1.13874	3.56E-05
MXAN_RS30810	MXAN_6361	hypothetical protein	35.09245	77.6905	-1.02796	0.000426
MXAN_RS31245	MXAN_6451	hypothetical protein	55.062	195.7092	-1.69008	5.46E-06
MXAN_RS31465	MXAN_6494	peptidase M23	46.5635	114.9565	-1.17052	3.57E-06
MXAN_RS31720	MXAN_6548	hypothetical protein	50.30406	178.6789	-1.70712	5.64E-07
MXAN_RS31725	MXAN_6549	transcriptional regulator, ROK family	40.65309	105.3761	-1.24337	7.11E-07

MXAN_RS31730	MXAN_6550	glycosyl hydrolase family 16	20.17034	55.87386	-1.34075	0.000108
MXAN_RS31735	MXAN_6551	peptide ABC transporter substrate-binding protein	29.66378	80.79531	-1.30553	2.91E-07
MXAN_RS31745	MXAN_6553	peptide ABC transporter permease	23.55466	60.32557	-1.2228	0.001097
MXAN_RS31755	MXAN_6555	beta-glucosidase	33.80345	83.15797	-1.16307	7.92E-05
MXAN_RS31760	MXAN_6556	hypothetical protein	70.96241	212.357	-1.44108	1.77E-07
MXAN_RS32035	MXAN_6618	hypothetical protein	28.47213	65.50154	-1.05771	2.88E-05
MXAN_RS32920	MXAN_6797	hypothetical protein	33.50977	80.54108	-1.15633	0.000195
MXAN_RS33880	MXAN_7000	hypothetical protein	86.62695	278.9601	-1.53091	1.54E-07
MXAN_RS34015	MXAN_7027	PAS domain-containing sensor histidine kinase	94.77377	181.4447	-0.79922	0.000436
MXAN_RS34315	MXAN_7088	hypothetical protein	18.00647	46.85835	-1.23417	0.000405

MXAN_RS34320	MXAN_7089	hypothetical protein	147.2378	325.8299	-1.0135	5.62E-05
MXAN_RS35310	MXAN_7297	STAS/SEC14 domain-containing protein	49.431	144.2351	-1.4169	5.11E-07
MXAN_RS35585	MXAN_7353	3-ketoacyl-ACP synthase	69.85785	134.224	-0.80564	0.000872
MXAN_RS35645	MXAN_7366	hypothetical protein	48.28053	96.7618	-0.86622	8.98E-05
MXAN_RS35770	MXAN_7393	Fis family transcriptional regulator	18.03947	45.76596	-1.19101	0.000404

Table S6. Strains and plasmids used in this study.

Strains or plasmids	Genotype or description	Source or references
Strains		
M. xanthus		
**DK1622**	Wild-type strains	D. Kaiser, University of Stanford
**LA(ΔlexA)**	*lexA* deletion mutant	This study
E. coli		
**DH5α(λpir)**	F^- φ80d*lacZ*ΔM15 Δ(*lacZYA-argF*) *U169 deoR recA1 endA1 hsdR17* ($r_k^- m_k^+$) *phoA supE44* λ^- *thi-1 gyrA96 relA1*	Takara
**BL21(DE3)**	Expression strain, λ(DE3 [lacI lacUV5-T7 gene 1 ind1 sam7 nin5]) [malB+]K-12(λS)	This study

Plasmids		
**pBJ113**	Gene replacement vector with KG cassette, Kan^r	Z.M. Yang, Virginia Tech
**pET15b**	*E. coli* expression vector, Amp^r	Novagen
**pET15b-lexA**	Gene *lexA* was inserted into *Nde*I/*Bam*HI sites of pET15b, Amp^r	This study

Table S7. Primers used in this study.

Primer name	Primer sequence (5’-3’)
**Gene knockout primers**	
MUT_4446_UF	TGACGGATCAGCCGCTCCTTGT
MUT_4446_UR	TCCTTGACGATGAAGCTGAGAAT
MUT_4446_DF	CGCACCCCGTAGTCCACCCCACCC
MUT_4446_DR	CCGCGGACGTGCTCGCCACCCTG
**Gene expression primers**	
LEXA_F	TACATATGGAAGAGCTCACGGAACGCC
LEXA_R	AAGGATCCGGGACGGGTGGGGTGGACTA
**RTPCR primers**	
RTPCR_nat_F	TGCGAGCCCTGAGTGACGA

RTPCR_nat_R	AGCCGAACTGGATGGACGAA
RTPCR_dnaE1_F	AACGACGAGAAGACCTGGGAGT
RTPCR_dnaE1_R	CCGTGCTTGCGGTTGATG
RTPCR_26715_F	GGGCAAGGGCACGCAGAAGT
RTPCR_26715_R	CGCCGTAGCAGAAGTGGCTCAC
RTPCR_recN_F	CTGGGTCTGCGGATTTCGA
RTPCR_recN_R	CACGGACGCCTCCTCACAA
RTPCR_lexA_F	ATGGAAGAGCTCACGGAACG
RTPCR_lexA_R	TCCTTGACGATGAAGCTGAG
RTPCR_yprA_F	GCCCATACCGGAAGAGGTGG
RTPCR_yprA_R	AGCGGCAGGTTGTAACAAAGG
RTPCR_uvrA_F	ATTCGCAGCCGCTTGTCC
RTPCR_uvrA_R	AGCAGCTTGCCGTTGTCG

RTPCR_gyrA_F	GCGACGAATCCACCAAGGA
RTPCR_gyrA_R	TCGGGCGTGCTCAACTCT
RTPCR_gyrB_F	TGCTGCTCACCTTCTTCTACCG
RTPCR_gyrB_R	CTTGGCGTTGCCCTTCGT
RTPCR_recA2_F	GGCAACGACGAGCCAATG
RTPCR_recA2_R	TCGGCGACGATGTGGAGA
RTPCR_lhr_F	GCGGGACGAGTTGATGGAGTG
RTPCR_lhr_R	GCGTTGGAAGAGGCTGAAGAGC
RTPCR_sbcC_F	AGGGCTATGCGTCCCACCG
RTPCR_sbcC_R	GCTCCGTCTCCAGCGTCTCC
RTPCR_sbcD_F	CGCGTTGAGCGAGCGGAAGA
RTPCR_sbcD_R	AGCACCTGATGCCGGTAGCC
